# Cerebral Atrophy in a Vitamin B12-deficient Infant of a Vegetarian Mother

**Published:** 2014-06

**Authors:** Celebi Kocaoglu, Fatih Akin, Hüseyin Çaksen, Saltuk Buğra Böke, Şükrü Arslan, Serhat Aygün

**Affiliations:** ^1^Konya Education and Research Hospital, Meram, Konya, Turkey; ^2^Necmeddin Erbakan University School of Medicine, Konya, Turkey

**Keywords:** Cerebral atrophy, Developmental regression, Failure to thrive, Macrocytic anaemia, Vitamin B12, Turkey

## Abstract

In developed countries, vitamin B12 (cobalamin) deficiency usually occurs in children, exclusively breastfed ones whose mothers are vegetarian, causing low body stores of vitamin B12. The haematologic manifestation of vitamin B12 deficiency is pernicious anaemia. It is a megaloblastic anaemia with high mean corpuscular volume and typical morphological features, such as hyperlobulation of the nuclei of the granulocytes. In advanced cases, neutropaenia and thrombocytopaenia can occur, simulating aplastic anaemia or leukaemia. In addition to haematological symptoms, infants may experience weakness, fatigue, failure to thrive, and irritability. Other common findings include pallor, glossitis, vomiting, diarrhoea, and icterus. Neurological symptoms may affect the central nervous system and, in severe cases, rarely cause brain atrophy. Here, we report an interesting case, a 12-month old infant, who was admitted with neurological symptoms and diagnosed with vitamin B12 deficiency.

## INTRODUCTION

Vitamin B12 or cobalamin deficiency is a rare and treatable cause of failure to thrive and delayed development in infants. In developed countries, the deficiency usually occurs in infants, exclusively breastfed ones whose mothers exhibit unrecognized pernicious anaemia or are vegetarian, causing low body stores of vitamin B12 in the infant at birth and inadequate amounts of the vitamin in the breastmilk (1). Because the only food source of infants is breastmilk, they are unlikely to consume animal products; so, vitamin B12 deficiency occurs easily. Signs and symptoms of vitamin B12 deficiency appear between the age of 4 and 12 months and include macrocytic anaemia, weakness, fatigue, failure to thrive, and irritability. Other common findings include pallor, glossitis, vomiting, diarrhoea, and icterus (1,2). Favourable response is achieved via vitamin B12 therapy, and especially neurological symptoms improve in a few days after the treatment. However, after the therapy, recovery is sometimes seen to vary from patient to patient remaining moderately or severely retarded (3,4). Therefore, efforts should be directed to prevent B12 deficiency in pregnant and breastfeeding women on vegan diets and their infants by administering B12 supplements. When preventive supplementation fails, healthcare providers should recognize and quickly treat the infant presenting with failure to thrive and delayed development (1). Thus, complete blood count, serum vitamin B12 level, and cranial magnetic resonance imaging (MRI) should be performed, and vitamin B12 supplementation should be started immediately.

## CASE REPORT

A 12-month old male infant from the province of Konya, Turkey, was referred to the Pediatrics Clinic of Konya Education and Research Hospital because of developmental regression and growth retardation in April 2012. Medical history taken from his mother revealed that he was born at term, weighing 3,500 g after uncomplicated pregnancy and delivery. He was exclusively breastfed, and his mother had been following a vegetarian diet for many years. The case showed normal developmental features up to 6 months: smiling at 2 months, controlling his head at 4 months, and starting to roll at 5 months. During the first 6 months of life, his body measurements were at the 25-50th percentile for weight and length and at the 50th percentile for head-circumference. When the child became 6 months old, his parents recognized that their baby stopped gaining weight and became less active.

**Figure 1.**
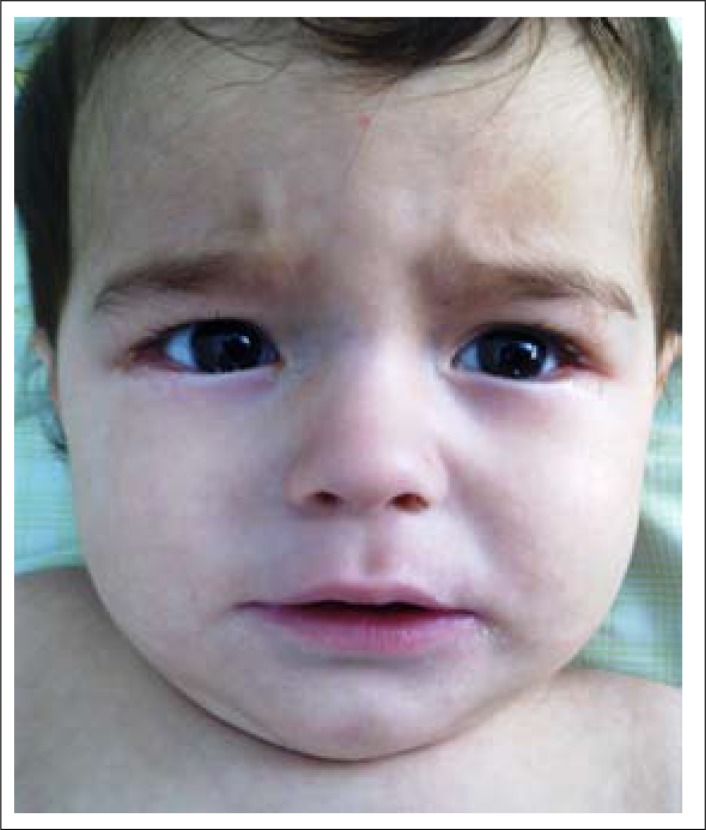
Facial appearence of the patient (marked rhagades around the angles of both eyelids and mouth)

**Figure 2.**
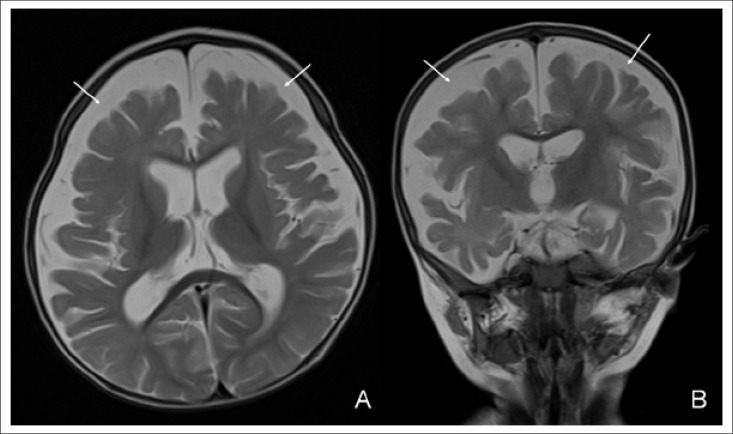
Initial MR images: axial (A) and coronal (B) T2-weighted images showed severe cerebral atrophy with enlargement of cortical sulci and subarachnoid spaces (arrows)

Although brisk reflexes and cranial nerve examination were normal on admission, he was lethargic, generally hypotonic, lacking smiling and failing to follow objects visually. His weight (8,600 g) and length (71 cm) were on the 10-25th percentile, and his head-circumference (45.5 cm) was on the 25th percentile. The results of general physical examination were normal; however, the case was determined to show rhagades around the angles of both eyelids and mouth as seen in the photo (Figure 1) [The baby's mother provided written approval to the authors to use the photograph in this case study]. Haemoglobin level, granulocyte and platelet counts were 8.8 g/dL, 6.02×10^3^/mm^3^, and 308×10^3^/mm^3^ respectively. Mean corpuscular volume, reticulocyte count, red blood cell count, and haematocrit were 97.3 fL (reference range 80-96 fL), 6×10^3^/mm^3^, 2.63×10^6^/mm^3^, and 21.3% respectively. The neutrophils were seen as hypersegmented. The serum cobalamin level was 117 pg/mL (reference range 211-911 pg/mL). Serum folate level was 13.85 ng/mL (reference range 3.1-20 ng/mL). Iron and ferritin levels, biochemical profile, and urine test results were normal, with normal blood and urine aminoacidography. Cortical atrophy and enlargement of subarachnoid space were evident on MRI scan (Figure 2). Vitamin B12, haemoglobin levels, and mean corpuscular volume of the mother were 232 pg/mL, 12.2 g/dL, and 104.5 fL respectively.

Megaloblastic anaemia due to vitamin B12 deficiency was diagnosed through a combination of clinical and laboratory findings, including clinical presentation, increased mean corpuscular volume (macrocytosis), hypersegmentation of neutrophils, and low vitamin B12 levels.

**Figure 3.**
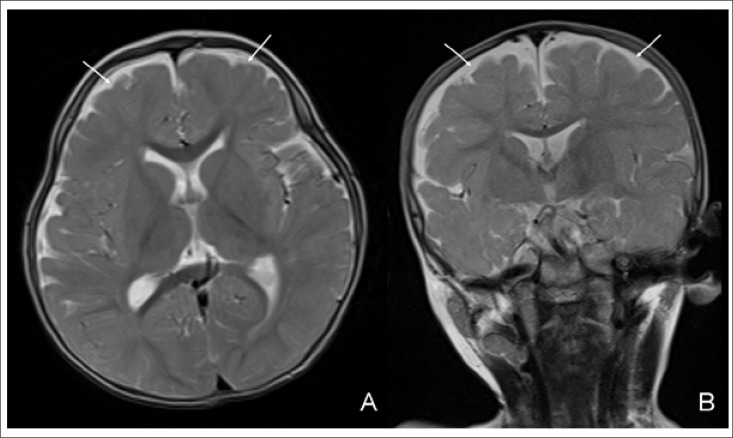
Control MR images after therapy. Axial (A) and coronal (B) T2-weighted images demonstrated recovery of cerebral atrophy. Subarachnoid space width was in normal range (arrows)

The case was given intramuscular injection of 1 mg of cobalamin and displayed a prompt neurological recovery. Three days after the first injection, he was smiling again and was neither lethargic nor hypotonic any longer. Haematologic values improved at the second week of the treatment. His parents were pleased because the case had a great improvement in his mental and motor development. Thus, the communication skills were improved with parents, the appetite became ameliorated, and he could control his head again. He started walking when he was 18 months old. Control cranial MRI performed three months after the initiation of therapy demonstrated recovery of cerebral atrophy. Subarachnoid space width was in normal range (Figure 3). With his neurologic improvement and normal haematologic values, cobalamin therapy was discontinued at the age of 18 months.

## DISCUSSION

Vitamin B12 deficiency is probably the second most common vitamin deficiency, causing anaemia. It is a megaloblastic anaemia, with high mean corpuscular volume and typical morphological features, such as hyperlobulation of the nuclei of the granulocytes (5). In developing countries, vitamin B12 deficiency constitutes a significant problem (6). A study from Turkey revealed that nearly 40% women of reproductive age had vitamin B12 deficiency (7). In addition to insufficient dietary vitamin B12 intake, such a deficiency may be due to malabsorption syndrome of cobalamin or pernicious anaemia.

Because of the lack of vitamin B12 synthesization in humans and its existence in only animal products, vegetarian, vegan or macrobiotic diets may lead to vitamin B12 deficiency (8). Recommended dietary intake of vitamin B12 for adults is 2.4 μg/day; 2 to 3 mg of vitamin B12 is stored primarily in the liver (2). Therefore, several years of dietary deficiency are necessary before the condition is clinically apparent. Daily requirements of vitamin B12 for children and adolescents range from 0.4 to 2.4 μg, and its storage is around 25 μg (9). Human foetus accumulates 0.1 to 0.2 μg of vitamin B12 per day. Dietary intake of vitamin B12 should increase from 2 to 2.2 μg/day during pregnancy (10). Cobalamin is actively transported across the placenta in foetuses of cobalamin-deficient mothers, and infants of such mothers are haematologically normal at birth. However, their cobalamin stores are low, and because the level of cobalamin in their mothers’ breastmilk corresponds closely with those in their sera, the scene is set for the development of cobalamin deficiency with the growth of infants, if breastfeeding is the only choice of diet. During the first six weeks of life, a considerable decrease is seen in the infants’ serum cobalamin level. This may reflect the efficient use of cobalamin in a growing organism, combined with marginal body stores and an inadequate supply (11). When evaluating the clinical and laboratory findings of the patient, we consider that the condition was related to vitamin B12 deficiency. Upon questioning the history in detail, the patient's mother reported to be a vegetarian and to receive no vitamin B12 supplementation during the reference pregnancy. Although the mother's haemoglobin level was within normal limits, serum cobalamin level was found to be close to the lower limits of normal and mean corpuscular volume to be higher. Therefore, it was considered that vitamin B12 storage of the mother was limited, and the mother failed to transfer enough vitamin B12 during the pregnancy and breastfeeding period. As a result, baby's development that was initially normal ceased due to wasting vitamin B12 storage, and the present clinical feature emerged.

Pathophysiology of the neurological impairment relating to B12 vitamin deficiency still remains unclear. Dror *et al*. (12) reported several theories listed below regarding the mechanisms by which vitamin B12 deficiency causes neurological impairment:

1. Delayed myelination or demyelination
2. The ratio of altered S-adenosylmethionine to S-adenosylhomocysteine
3. Tumour necrosis factor-α and epidermal growth factor imbalanc
4. Accumulation of lactate

Myelination defects due to vitamin B12 deficiency can have significant effects on central nervous system functioning by altering the speed of conduction in multiple systems. For example, slower conduction in the auditory and visual systems can interfere with learning and social interaction. So, many brain systems are myelinated during the early developmental period. The acquisition of cognitive skills coincides with the pattern of central nervous system myelination. Therefore, retardation of myelination of the brain in infancy leads to delayed acquisition of cognitive skills, and brain atrophy leads to regression of these skills (9). Despite being 12 months old, our case was lethargic, not smiling, and failed to follow the objects visually.

Symptoms and signs of vitamin B12 deficiency appear between the age of 4 and 12 months and include failure to thrive, lethargy, hypotonia, and arrest or regression of developmental skills. It is a rare cause of epileptic seizures during infancy. As in our case, neurological symptoms seem to affect central nervous system, and, in severe cases, brain atrophy develops (13). Approximately half of the affected infants exhibit abnormal movements, including tremor, myoclonus, and choreoathetoid movements. These involuntary movements usually recover within a few days after the treatment (3).

In literature, due to difficulties relating to follow-ups, limited data exist on long-term development after severe neurological changes in infantile vitamin B12 deficiency. Low intelligence quotient (IQ) and a psychomotor and linguistic delay are reported to be associated with long-term dysfunctions after prolonged vitamin B12 deficiency in infants (2). The long-term prognosis depends on overall duration of deficiency and severity of symptoms rather than serum levels of vitamin B12 or haemoglobin values on admission. It seems that infants diagnosed and treated before one year of age have more favourable neurological outcome than those treated at a later period. It should be remembered that the initial improvement after the treatment may not result in favourable outcome in the long term (4). In our patient, prompt neurological recovery was observed. Three days after the first injection, he was smiling again and was neither lethargic nor hypotonic. At the sixth month of treatment, he started walking when he was 18 months old. Control cranial MRI performed three months after the initiation of therapy demonstrated recovery of cerebral atrophy.

Findings from the present case study suggest that vitamin B12 deficiency should be considered in the aetiological diagnosis of neurological symptoms in infants, especially in the presence of megaloblastic anaemia and developmental delay. Healthcare providers should take into account that patients with vitamin B12 deficiency can present unexpected signs and symptoms, such as developmental delay, cerebral atrophy, and subdural effusion as seen in our patient.

### Conclusions

Vitamin B12 deficiency is a rare but treatable cause of neurological disorders, anaemia, and failure to thrive in infants exclusively breastfed by vitamin B12-deficient mothers. Recognition of the neurological symptoms of infantile vitamin B12 deficiency may allow early diagnosis and appropriate treatment. Because of the importance of vitamin B12 in the development of the foetal and neonatal brain, vegetarian and vegan mothers should be aware of the severe and not fully-reversible damages caused by insufficient nutritional intake of vitamin B12 during pregnancy and lactation. Therefore, efforts should be directed to prevent its deficiency in pregnant and breastfeeding women on vegan diets and their infants. It is also important to take the nutritional history of both infants and their mothers for the early prevention and treatment. To increase the dietary intake of vitamin B12, the diet should be rich in foods of animal origin, such as dairy products, red meat, egg and fish. Recommended dietary intake of B12 should increase from 2 to 2.2 µg/day during pregnancy. With early awareness, potentially irreversible neurologic damages can be prevented.
